# No Impact of COVID-19 Pandemic on Early Mortality for Thyroid Cancer in the US. Comment on Lee et al. Impact of the COVID-19 Pandemic on Thyroid Cancer Surgery. *Curr. Oncol.* 2024, *31*, 3579–3590

**DOI:** 10.3390/curroncol31100466

**Published:** 2024-10-17

**Authors:** Riccardo Nocini, Giuseppe Lippi, Camilla Mattiuzzi

**Affiliations:** 1 Unit of Otolaryngology, Head and Neck Department, University of Verona, 37134 Verona, Italy; riccardo.nocini@aovr.veneto.it; 2 Section of Clinical Biochemistry, University of Verona, 37134 Verona, Italy; 3 Medical Direction, Rovereto Hospital, Provincial Agency for Social and Sanitary Services (APSS), 38068 Rovereto, Italy; camilla.mattiuzzi@apss.tn.it

**Keywords:** mortality, thyroid, cancer, COVID-19

Thyroid cancer is relatively rare in the general population compared to other malignancies, but its incidence appears to have increased in recent decades. In a recent article, Lee at al. [1] conducted a search of the US National Cancer Database to investigate whether major disruptions in cancer diagnosis during the coronavirus disease 2019 (COVID-19) pandemic may have affected the early detection and management of thyroid cancer. The authors found that despite a decrease in the overall number of diagnoses for this malignancy, resource allocation remained almost unchanged during the pandemic. For this reason, we designed this study to investigate whether early mortality from thyroid cancer in the US has changed during the COVID-19 pandemic.

We performed an electronic search using the US Centers for Disease Control and Prevention (CDC) Wonder (Wide-Ranging, Online Data for Epidemiologic Research) database, a nationwide repository which contains a rich ad hoc query system for the analysis of public health data, especially unique causes of death, between the years 2018 and 2022 [2]. The information contained in the registry is based on death certificates for the entire US resident population. We defined our search setting “year” (between 2018 and 2022) as the first variable, combined with the ICD-10 code for all thyroid cancers (C73: malignant neoplasm of thyroid gland). The data were retrieved in the form of age-adjusted death rate (×100,000) and 95% confidence interval (95%CI). Statistical significance throughout the study period was evaluated with a one-way analysis of variance (ANOVA) and a Tukey post hoc test, according to the total number of deaths for thyroid cancer and the mean age-adjusted death rate and its standard error (SE), using StatPages (Interactive Statistical Calculation). The statistical significance was set at *p* < 0.05. This study was conducted in accordance with the Declaration of Helsinki and the terms of the relevant local legislation. This study was exempt from Institutional Review Board review as CDC WONDER is a de-identified and publicly available database.

The results of our analysis are shown in Figure 1. According to the WONDER database, the age-adjusted mortality rate (×100,000) for thyroid cancer in the US was 0.503 in 2018 (95%CI, 0.481–0.525), 0.506 in 2019 (95%CI, 0.483–0.528), 0.483 in 2020 (95%CI, 0.461–0.505), 0.510 in 2021 (95%CI, 0.487–0.532) and 0.517 in 2022 (95%CI, 0.495–0.539). Overall, the one-way ANOVA (f = 1.33; *p* = 0.255) found no statistical differences during the observation period. In the Tukey post hoc test analysis, no significant difference was found in the multiple comparison of the individual years during the entire study period (Table 1).

The results of our analysis provide additional insights into the impact of the COVID-19 pandemic on thyroid cancer outcomes and complement the results already presented by Lee et al. [1]. Consistent with their finding that patients with thyroid cancer had similar treatment times before (i.e., in 2019) and during the early phase of the pandemic (i.e., in 2020), we also failed to find a significant difference in the age-adjusted mortality rate between 2018 and 2022. This evidence is consistent with other previous reports, such as that of Kathuria-Prakash et al. [3], who found that initial disease burden and extent, as well as therapy and its response, showed no significant association with the severity of COVID-19, or the report of Sahin et al. [4], which showed that COVID-19 disease does not cause an enhanced risk of death in patients with a history of thyroid cancer [4].

Therefore, we can conclude that the possibility of timely treatment of thyroid cancer with prioritization of advanced-stage patients during the COVID-19 pandemic has allowed early mortality for thyroid cancer to remain at a level comparable to that before the pandemic. Nonetheless, we cannot discount that COVID-19 may still have implications for thyroid cancer patients, as it can affect their outcomes through various mechanisms [5,6], such as cytokine storm, which can negatively impact multiple organs, including the thyroid; immune system dysregulation, immunosuppression and the resulting enhancement of tumor immune evasion, which can be caused by the virus or induced by cancer or COVID-19 treatments (e.g., chemotherapy, steroids); autoimmunity, as some cancer patients are known to develop anti-thyroid antibodies after COVID-19; and anxiety and stress, which may influence cancer progression via neuroendocrine and immunological mechanisms.

## Figures and Tables

**Figure 1:**
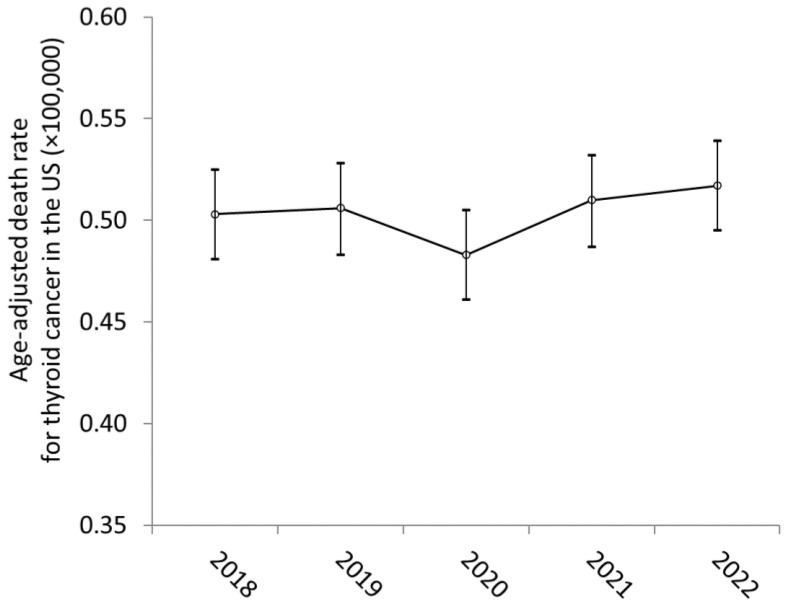
Mortality for thyroid cancer in the US between 2018 and 2022. The data are shown as the age-adjusted death rate ×100,000. The statistical analysis is reported in the manuscript.

**Table 1:** Tukey post hoc test analysis (*p*-values) for between-year comparison of age-adjusted mortality for thyroid cancer in US between 2018 and 2022.

Years	2019	2020	2021	2022
2018	0.999	0.713	0.992	0.894
2019	-	0.590	0.999	0.953
2020	-	-	0.414	0.182
2021	-	-	-	0.991
